# Population Genetic Analyses of *Botrytis cinerea* Isolates From Michigan Vineyards Using a High-Throughput Marker System Approach

**DOI:** 10.3389/fmicb.2021.660874

**Published:** 2021-04-20

**Authors:** Rachel P. Naegele, Jeff DeLong, Safa A. Alzohairy, Seiya Saito, Noor Abdelsamad, Timothy D. Miles

**Affiliations:** ^1^Crop Diseases, Pests and Genetics Unit, United States Department of Agriculture, Agricultural Research Service, San Joaquin Valley Agricultural Sciences Center, Parlier, CA, United States; ^2^Small Fruit and Hop Pathology Laboratory, Department of Plant, Soil and Microbial Sciences, Michigan State University, East Lansing, MI, United States; ^3^Commodity Protection and Quality Unit, United States Department of Agriculture, Agricultural Research Service, San Joaquin Valley Agricultural Sciences Center, Parlier, CA, United States

**Keywords:** population genetics, fungicide resistance, amplicon sequencing, microsatellite, genetic diversity, gray mold

## Abstract

As sequencing costs continue to decrease, new tools are being developed for assessing pathogen diversity and population structure. Traditional marker types, such as microsatellites, are often more cost effective than single-nucleotide polymorphism (SNP) panels when working with small numbers of individuals, but may not allow for fine scale evaluation of low or moderate structure in populations. *Botrytis cinerea* is a necrotrophic plant pathogen with high genetic variability that can infect more than 200 plant species worldwide. A panel of 52 amplicons were sequenced for 82 isolates collected from four Michigan vineyards representing 2 years of collection and varying fungicide resistance. A panel of nine microsatellite markers previously described was also tested across 74 isolates from the same population. A microsatellite and SNP marker analysis of *B. cinerea* populations was performed to assess the genetic diversity and population structure of Michigan vineyards, and the results from both marker types were compared. Both methods were able to detect population structure associated with resistance to the individual fungicides thiabendazole and boscalid, and multiple fungicide resistance (MFR). Microsatellites were also able to differentiate population structure associated with another fungicide, fluopyram, while SNPs were able to additionally differentiate structure based on year. For both methods, AMOVA results were similar, with microsatellite results explaining a smaller portion of the variation compared with the SNP results. The SNP-based markers presented here were able to successfully differentiate population structure similar to microsatellite results. These SNP markers represent new tools to discriminate *B. cinerea* isolates within closely related populations using multiple targeted sequences.

## Introduction

*Botrytis cinerea* is a necrotrophic pathogenic fungus that infects hundreds of plant species (Alfonso et al., 2000; Ma and Michailides, 2005; Williamson et al., 2007; Fillinger, 2016) including economically important crops such as fruits, ornamentals, and vegetables (Elad et al., 2004; Leroux, 2007). *B. cinerea* causes gray mold, a major worldwide destructive disease that results in significant yield loss of grapevines in the field and postharvest (Campia et al., 2017; Rupp et al., 2017b; Saito et al., 2019; Alzohairy et al., 2020). *B. cinerea* infects all grapevine plant parts, though fruit rot, known as botrytis bunch rot (preharvest) and gray mold (postharvest), is the most common (Gabler et al., 2003; Elmer and Michailides, 2007; Saito et al., 2019).

Control of gray mold is dependent on regular applications of synthetic fungicides. Several Fungicide Resistance Action Committee (FRAC) classes are available to control bunch rot, including quinone outside inhibitors (QoIs), benzimidazole, phenylpyrroles, succinate dehydrogenase inhibitors (SDHIs), anilinopyrimidines (APs), dicarboximides, and hydroxyanilides (Leroux, 2004). However, *B. cinerea* is considered a difficult pathogen to control due to its rapid spread by wind (Holz et al., 2007) and the high genetic variability (Leroux et al., 2002) that allow the pathogen to develop resistance against applied synthetic fungicides (Rupp et al., 2017b). In grapes, fungicide resistance has been reported in different countries worldwide (Leroch et al., 2011; Angelini et al., 2014; Panebianco et al., 2015; Yin et al., 2015) and in the US including Michigan (Alzohairy et al., 2020) and California (Saito et al., 2019; Avenot et al., 2020; Delong et al., 2020). An increasing number of isolates with resistance to not only a single fungicide but also to multiple fungicides of different chemical classes have been reported (Leroch et al., 2013; Fernández-Ortuño et al., 2015; Saito et al., 2019; Alzohairy et al., 2020; Delong et al., 2020). Fungicide resistance frequencies have been shown to differ between years, crop hosts, and locations (Fernández-Ortuño et al., 2015; Delong et al., 2020; Kozhar et al., 2020).

The genus *Botrytis* is highly genetically diverse with more than 30 species that differ in morphology, ecology, biology, and host range (Walker, 2016). The genus *Botrytis* was generally considered as a single complex species until the late 1990s when it was subdivided into two clades, one clade contains *Botrytis* spp. that infect mostly monocots and some dicots, while the second clade contains *Botrytis* spp. that infect a wide host range of eudicots; *B. cinerea* falls under this second clade (Staats et al., 2005). Population structure and genetic variations have been studied in *B. cinerea* populations, and new morphologically identical or similar species were identified. Recently a number of cryptic species causing gray mold that lives in sympatry with the *B. cinerea* complex have been identified on a variety of hosts (Li et al., 2012; Saito et al., 2016; Dowling et al., 2017; Rupp et al., 2017b; Harper et al., 2019). These new cryptic species are more likely considered as host or region specific. Formerly known as *B. cinerea* Group I, *B. pseudocinerea* isolates are morphologically identical and live in sympatry with *B. cinerea* (Fournier et al., 2005; Walker et al., 2011). Genetic polymorphisms in transposable element presence and a group of genes including Bc-*hch*, erg27, sdh, and cyp51 between *B. cinerea* and *B. pseudocinerea* provided evidence for the differentiation of *B. pseudocinerea* as a new species (Fournier et al., 2003; Walker et al., 2011; Plesken et al., 2015). Within *B. cinerea sensu stricto*, a large variability in genetic and phenotypic diversity, and host specialization have also been observed (Corwin et al., 2016; Mercier et al., 2019; Soltis et al., 2019; Meng et al., 2020).

Generally, the population structure in *B. cinerea* was detected to vary between different hosts (Fournier and Giraud, 2008; Walker et al., 2015) or year (Walker et al., 2015; Delong et al., 2020), while less or no variation was detected at the region level (Fournier and Giraud, 2008; Karchani-Balma et al., 2008; Esterio et al., 2011; Wessels et al., 2013; Walker et al., 2015). On the other hand, Muñoz and Moret (2010) found that genetic diversity was high, and population structure varied when comparing isolates of *B. cinerea* at a continent level. The molecular marker method that is used to investigate genetic differences can also contribute to the observed genetic differences (Walker et al., 2011).

Various molecular markers have been used to investigate the genetic variability and population structure in *B. cinerea*. Fournier et al. (2003) developed a PCR-RFLP method that differentiated *B. pseudocinerea* from the *B. cinerea* complex based on the polymorphism detected in the Bc-*hch* gene. Other genes that are *Botrytis* spp. specific were used to differentiate different populations on table grape and blueberry using Sanger sequencing (Staats et al., 2005, 2007; Saito et al., 2016). Recently, DeLong et al. developed a set of microsatellite markers spanning the genome to characterize *Botrytis* populations (2020). Commonly, the presence/absence of TEs is used for the determination of *Botrytis* sp. population diversity (Kretschmer and Hahn, 2008; Fekete et al., 2012; Wessels et al., 2016; Hu et al., 2018). Previous studies that used TEs or microsatellites as markers for population differentiation showed that different hosts can be dominated by different populations such as grape and pomegranate, and were dominated by *transposa* isolates that have both TEs (Váczy et al., 2008; Johnston et al., 2014; Delong et al., 2020; Testempasis et al., 2020). The fungicide resistance profile is different between different strains of *Botrytis* (Martínez et al., 2008; Leroux et al., 2010; Johnston et al., 2014; Delong et al., 2020). However, studies of population variability in relation to fungicide resistance profile showed limited to no association with the population structure (Wessels et al., 2016; Campia et al., 2017; Hu et al., 2018; Delong et al., 2020).

Several studies in other systems have compared the use of single-nucleotide polymorphism (SNP) or sequencing-based markers and microsatellite markers to describe population structure and diversity (Fischer et al., 2017; Lemopoulos et al., 2018). In one such study, SNP diversity estimates and microsatellite heterozygosity in *Arabidopsis* were not significantly correlated, but genetic differentiation among populations was correlated (Fischer et al., 2017). Similarly in trout, 16 microsatellites performed similarly to >4,000 SNPs at measuring genetic differentiation, but SNPs were more accurate at estimating individual level heterozygosity (Lemopoulos et al., 2018). However, similar studies have found that studies, where a low number of SNPs >300 were used, had lower power than microsatellites (Vali et al., 2008). Yet most of these studies involved animals or plants with larger plant genomes than *Botrytis* (Ciani et al., 2013; Singh et al., 2013). A study on *Plasmodium vivax*, a malarial parasite, demonstrated that 146 high-quality SNPs using an amplicon sequencing approach were more informative than microsatellite markers (Fola et al., 2020).

Because different *Botrytis* sp. can exhibit differences in fungicide resistance profile, it is critical to understand the pathogen population structure in different environments. This will allow development of better disease management schemes. There is no information about *B. cinerea* population structure and genetic diversity in Michigan grapevine. Therefore, this is the first study to investigate the genetic diversity of the *B. cinerea* population in Michigan grapes in combination with fungicide sensitivity phenotypic characteristics. Our objectives were to study the population structure of MI isolates of *B. cinerea* related to year, location, fungicide resistance, and to compare the use of microsatellite and amplicon-based sequencing SNP strategies to quantify genetic diversity and population structure.

## Materials and Methods

### Isolates and DNA Extractions

A total of 82 *B. cinerea* isolates were collected from four Michigan vineyards, three southwest and one northwest Michigan locations (Supplementary Table 1). The “West” Michigan vineyard location represents *B. cinerea* isolates recovered from a *Vitis* interspecific hybrid (cv. Vignoles), and “Southwest” Michigan vineyards 1 and 2 are also hybrids recovered from cultivars Vignoles and Aurora, respectively. Finally, the “Northwest” Michigan samples were recovered from symptomatic *Vitis vinifera* (cv. Reisling).

These locations were sampled in both 2014 and 2018, where 42 and 40 samples were collected in 2014 and 2018, respectively, with at least eight isolates per location. Throughout the study, isolates were maintained on 20% clarified V8 agar media (100 ml of V8 juice, 1 g of CaCO_3_, 10 g of agar, and 400 ml of distilled water) at room temperature. All isolates were previously evaluated for fungicide resistance against eight fungicides with seven different chemical classes (Alzohairy et al., 2020). For each isolate, a multiple fungicide resistance (MFR) value was determined based on the total number of fungicides an isolate demonstrated resistance toward. For DNA extraction and quantification, we followed Alzohairy et al. (2020); in brief, mycelia were collected from 1- to 2-week-old cultures. Mycelia were lyophilized, then approximately 5 mg of tissue was ground using a tissuelyser (Qiagen, Valencia, CA). Automated DNA extraction was performed using a MagMax plant DNA isolation kit (ThermoFisher 192 Scientific, Waltham, MA) and processed on the KingFisher Flex purification system (ThermoFisher Scientific). For DNA quantification, we used two methods: first, DNA samples were processed with a Qubit 1X dsDNA HS assay kit (ThermoFisher Scientific), then DNA concentrations were quantified by a Qubit 4 fluorometer (ThermoFisher Scientific); second, DNA samples were processed using PicoGreen Quant-iT DNA reagent and kits (ThermoFisher Scientific), then DNA concentrations were determined using the Synergy HTX multi-mode reader (Biotec, VT). Insufficient DNA was available for all 82 isolates for both microsatellite and SNP evaluation, and only 74 isolates were consistent between the two datasets.

### Multiplex PCR and Sequencing

#### Candidate Genes and Neutral Markers

Candidate genes were selected to screen for resistance-associated mutations in β-tubulin (*tub*), *sdhB*, cytochrome b (*cytb*), and keto-reductase (*erg27*), mating type (*Mat1-1* and *Mat1-2*), transposable element (*Flipper*), virulence (*Nep1* and *mrr1*), and species identification (*G3PDH*, *ITS*, *RPB2*, and *HSP60*; Supplementary Table 2). Previously published primer sets for diversity testing using SSRs were also included (Fournier and Giraud, 2008; Delong et al., 2020; Supplementary Table 2). Primers for candidate genes were designed using Primer3 software (Koressaar et al., 2009; Untergasser et al., 2012) and screened using the three-primer method (Delong et al., 2020). All markers were validated on a subset of 15 *Botrytis* isolates using the following 25 μl PCR reaction; 12.5 μl of 2× GoTaq Green Master Mix (Promega Corp.), 1 μl of 10 μM forward primer, 1 μl of 10 μM reverse primer, 1 μl of DNA template (50 ng/μl), and 9.5 μl of sterile double-deionized water (sddH_2_O). Reaction conditions were 95°C for 2 min followed by 30 cycles of denaturation at 95°C for 30 s, annealing at 60°C for 30 s, and extension at 72°C for 1 min, then a final extension at 72°C for 5 min. The PCR products were separated by electrophoresis at 65 V for 1.5 h on ethidium bromide stained (10 μg/ml) 1.5% agarose gel, then visualized under the UV light for validation. All primer sets successfully produced clear bands within the expected size range for the 15 isolates. Designed primers were aligned to the reference genome B05.10 (ASM83294v1 http://fungi.ensembl.org/Botrytis_cinerea/Info/Index) using Geneious Prime v 2020.2.1 to determine the expected SNP locations.

#### Design of Multiplex Primer Sets

Primers (microsatellite and candidate genes) were grouped in sets of five, six, or eight-plex for simultaneous amplification of *Botrytis* target genes. Grouping was based on the following parameters: (1) expected amplicon size, with at least 30-bp difference between each amplicon for clear validation by gel electrophoresis and (2) the same annealing temperature. Since the presence of multiple oligonucleotides in one PCR reaction could alter the efficiency of amplification, several combinations of primer concentrations (0.2–0.4 μM) were tested in parallel in a single-plex and in multiplex formats using the cycling conditions mentioned above. Amplicons were separated by electrophoresis at 50 V for 2 h on ethidium bromide-stained (10 μg/ml) 4% agarose gel and visualized under the UV light for validation of band presence.

#### Multiplex PCR and Samples Pooling

After primer validation, multiplex groups (five-, six-, or eight-plex) were used to amplify *Botrytis* target regions on all 82 isolates. For each multiplex PCR reaction, each isolate was amplified using QIAGEN Multiplex PCR Kit (Qiagen) as follows: 5 μl of 2× QIAGEN Multiplex PCR master mix (Qiagen), 1 μl of 10× primer mix, 2 μl of DNA template (50 ng/μl), and 2 μl of sddH_2_O. Reaction conditions were 95°C for 2 min followed by 30 cycles of denaturation at 95°C for 30 s, annealing temperature varied between 50 and 60°C (based on primer design) for 30 s, and extension at 72°C for 1 min and, then, a final extension at 72°C for 5 min. For sample DNA pooling, the multiplex PCR products were grouped by isolates as follows: for each isolate, 5 μl from each multiplex group was transferred to new PCR plates to end up having a total of 15 μl of DNA per isolate, representing three multiplex groups (5 μl each). DNA was diluted 1:10 using sddH_2_O in PCR plates for quantification using Qubit dsDNA HS Assay Kit (ThermoFisher Scientific) following the manufacture procedure. Concentrations were adjusted to 2–10 ng/μl of DNA and submitted to the Research Technology Support Facility (College of Natural Science, Michigan State University) for sequencing using a 250-bp paired-end MiSeq.

### Single-Nucleotide Polymorphism Processing and Alignments

Quality control, processing, read alignments, and SNP calling were completed using the Galaxy bioinformatics server.^1^ Reads were trimmed using Trimmomatic (v0.38.0) with paired-end adapter trimming for Illumina MiSeq and HiSeq adapters. Reads shorter than 30 bp were removed, and read quality was assessed using a sliding window of 4 bp with a quality ≥20. Overrepresented sequences, GC content, and read quality were visualized using FastQC. Single reads (reads without a corresponding mate) were not retained for downstream analyses. Mapping was performed using Bowtie2, read groups (Sam/Bam format) were automatically set, and alignments were based on the sensitive local parameters to the *B. cinerea* reference genome (Van Kan et al., 2017). Unaligned reads were output to a separate file. Sam files were merged for downstream analysis. Single-nucleotide polymorphisms (SNPs) and insertions/deletions (indels) were identified for each isolate using FreeBayes. Indels were left aligned, and 60% of the observations were required for an alternate allele to be suggested within an individual. The variant call format (VCF) file was further filtered using TASSEL 5 (v20180222) to remove sites present in fewer than 10 individuals. The resulting VCF with 496 SNPs was used for all downstream analyses. An SNP was considered to be associated with a primer set, if the variant fell within the mapped boundaries of the forward and reverse primer visualized with Geneious Prime.

### Microsatellite Genotyping

Published microsatellites were evaluated across 74 isolates using a three-primer method as previously described (Schuelke, 2000; Delong et al., 2020; Supplementary Table 1). PCR was carried out in 25-μl reactions with 12.5 μl of 2× GoTaq Clear Master Mix (Promega Corp. Madison, WI), 0.75 μl of 10 μM forward primer, 1.25 μl of 10 μM 5' 6FAM-labeled M13 tag primer, 2.0 μl of 10 μM reverse primer (Invitrogen Inc. Carlsbad, CA), 7.5 μl of sddH_2_O, and 1.0 μl of approximately 25.0 ng/μl of DNA template. Reaction conditions were 95°C for 2 min followed by 29 cycles of denaturation at 95°C for 30 s, annealing (temperature varied between 50 and 60°C, specific to optimal temperature based on primer design) for 30 s, extension at 72°C for 30 s, followed by a final extension at 72°C for 5 min. One microliter of PCR product was added to 0.3 μl of GeneScan 500 LIZ-labeled size standard and 9.7 μl of Hi-Di formamide (Applied Biosystems Inc. Foster City, CA). Amplicons were denatured by incubation at 95°C for 5 min and immediately placed on ice. Fragment analysis was conducted on an Applied Biosystems 3730xl 96-capillary DNA Analyzer. Geneious Prime v.11.0.3 (Kearse et al., 2012) was used to determine allele sizes based on electropherograms. Isolates with >33% missing data (missing a peak or a failed reaction) were removed from the dataset. Microsatellite data were clone corrected using *Poppr* (v 2.8.6) implemented within R (v 4.0.2; R Development Core Team, 2012). Seventy isolates were retained for use with all downstream analyses.

### Genetic Diversity and Population Structure

For both the SNP and the microsatellite data sets, genetic diversity, and analysis of molecular variance (AMOVA) were calculated for the population based on location, year, and fungicide resistance using *Poppr*. Insufficient resistant or sensitive isolates were available for fludioxonil or pyraclostrobin, and thus, AMOVAs were not conducted for these fungicides. For the microsatellite data, MFRs for 1, 2, 3, 4, 5, and 6+ were compared due to an insufficient number of isolates in the 6 and 7 categories. Genetic distance trees were generated using a UPGMA tree based on Provesti’s distance (Parada-Rojas and Quesada-Ocampo, 2018).

## Results

### Single-Nucleotide Polymorphism-Based Diversity

**Figure 1:**
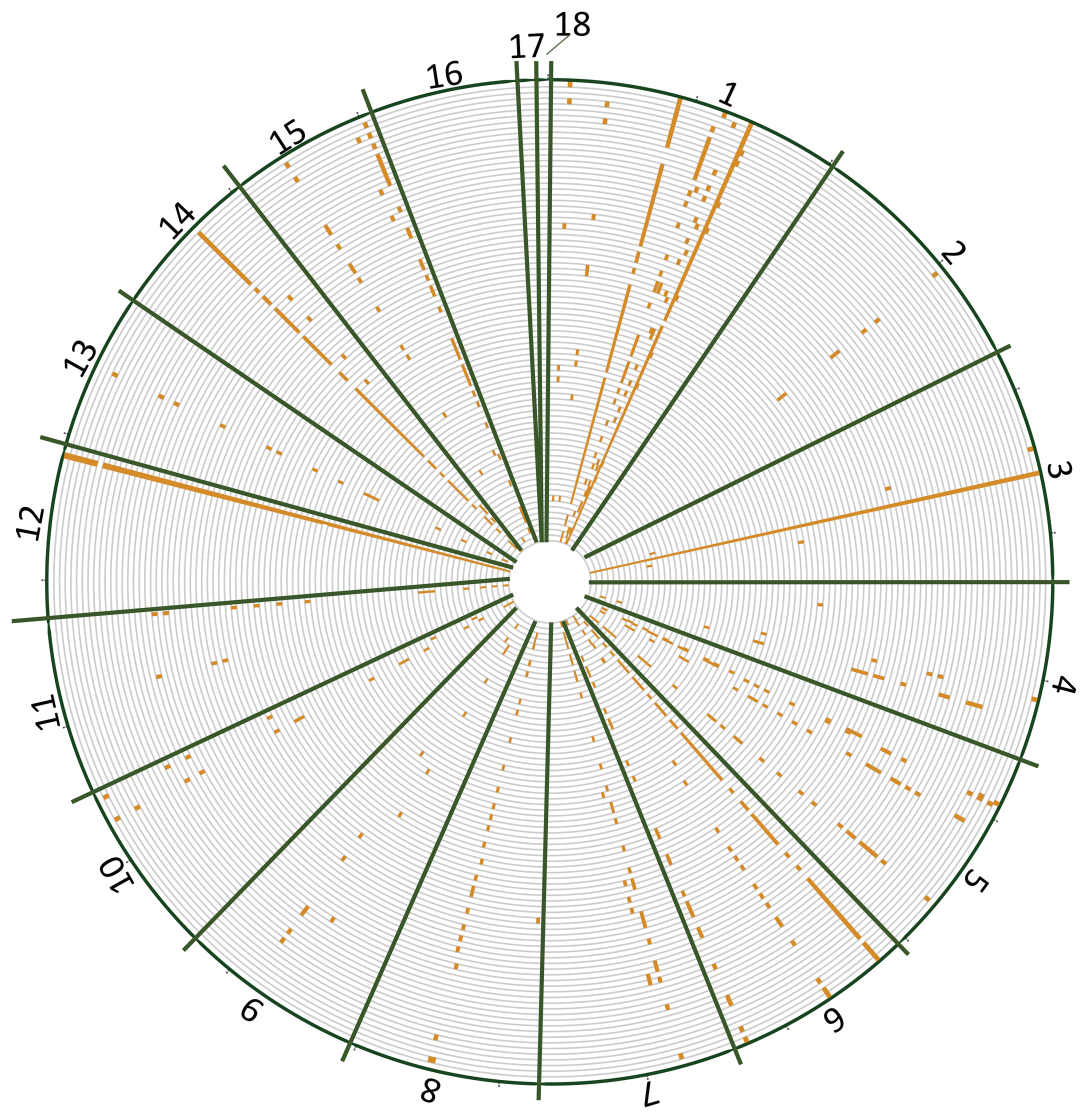
Single-nucleotide polymorphism (SNP) distribution (orange ticks) across each chromosome (green bars) of the *Botrytis cinerea* genome for *B. cinerea* isolates collected from Michigan vineyards.

The number of reads per isolate ranged from 14,000 to 32,000 and the percentage of reads mapped to the reference genome ranged from 95 to 99.92% (Supplementary Table 3). Overrepresented sequences ranged from 7 to 11 sequences per isolate. On average, each isolate had 23,844 reads with nine overrepresented sequences and 97% of reads mapping to the reference genome. When BLASTed against the *Botrytis* genome, unaligned reads were able to be aligned or were determined to be *Botrytis* but not in the nuclear reference genome (e.g., *Cyt b* and *Mat1-2*). SNP distribution across the genome ranged from three SNPs on chromosome 11 to 101 SNPs on chromosome 1 with a total of 496 SNPs detected post filtering used for subsequent analyses (Table 1). No SNPs were identified on chromosomes 16, 17, or 18, consistent with primer design (Figure 1). The greatest number of positional variants was detected for products associated with primer sets NEPO5, Bc_pop22, alpha 1, Flip3, Bc_pop16, Bc_pop59, Bc_pop82, and *mrr1* (Table 2).

**Table 1:** Single-nucleotide polymorphism (SNP) distribution across the *Botrytis* genome.

Chromosome	Number of SNPs
1	104
2	8
3	16
4	17
5	77
6	46
7	19
8	41
9	15
10	36
11	3
12	32
13	20
14	39
15	23
16	-
17	-
18	-
Total	496

**Table 2:** Mapped position of primer sets relative to the *Botrytis cinerea* reference genome and the total number of positional SNPs located within the product boundaries.

Primer	Gene name	Purpose	Chromosome	Starting position of primer	End position of primer	No. of SNPs^x^
HMG3	MAT1-2 HMG	Mating type	1	Not in reference	2,909,239	9
HMG2	MAT1-2 HMG	Mating type	1	Not in reference	2,629,868	1
NEPO5	NEP1	Virulence	6	2,351,497	2,351,754	17
Bc3		Microsatellite	1	277,529	277,750	7
N_Bc_pop22	BC1G_00215	Microsatellite	1	2,591,465	2,591,645	15
N_Bc_pop72	BC1G_00283	Microsatellite	1	2,431,994	2,432,202	4
N_Bc_pop90	BC1G_00324	Microsatellite	1	2,341,658	2,341,889	0
Alpha1	MAT1-1 α	Mating type	1	815,137	815,347	8
Flip3	Flipper	Transposable element	14,12	1,589,025, 8,353	15,889,046, 8,550	10, 18
N_Bc_pop17	BC1G_15884	Microsatellite	10	2,266,952	2,267,104	2
N_Bc_pop53	BC1G_03602	Microsatellite	10	1,923,167	1,923,342	4
N_Bc_pop78	BC1G_13432	Microsatellite	10	1,225,628	12,258,899	3
N_Bc_pop81	BC1G_05124	Microsatellite	10	446,603	446,476	5
N_Bc_pop48	BC1G_04625	Microsatellite	11	1,297,541	1,297,660	1
N_Bc_pop89	BC1G_13854	Microsatellite	12	2,288,599	2,288,797	4
MS5	MS547	Species Id	12	1,034,628	1,034,861	2
N_Bc_pop16	BC1G_04929	Microsatellite	13	1,213,165	1,213,421	15
N_Bc_pop35	BC1G_06422	Microsatellite	13	2,027,869	2,028,138	1
N_Bc_pop36	BC1G_11998	Microsatellite	14	1,261,613	1,261,855	5
N_Bc_pop38	BC1G_11999	Microsatellite	14	1,264,180	1,264,320	1
RPB1	RPB2	Species Id	14	703,587	730,818	13
N_Bc_pop34	BC1G_11591	Microsatellite	15	1,868,719	1,868,992	1
N_Bc_pop57	BC1G_13682	Microsatellite	15	655,984	656,108	1
G3P4	G3PDH	Species Id	15	731,751	731,978	1
N_Bc_pop85	BC1G_08081	Microsatellite	2	1,983,066	1,983,741	2
N_Bc_pop12	BC1G_11679	Microsatellite	3	2,515,919	2,516,035	2
N_Bc_pop62	BC1G_06127	Microsatellite	3	2,148,252	2,148,353	2
N_Bc_pop79	BC1G_06541	Microsatellite	3	907,286	907,387	1
Bchch2	Bc-hch	Group II Vs Group I	3	1,379,221	1,379,288	6
N_Bc_pop13	BC1G_04200	Microsatellite	4	1,606,579	1,606,693	3
N_Bc_pop29	BC1G_10612	Microsatellite	4	556,235	556,349	1
N_Bc_pop87	BC1G_03782	Microsatellite	4	1,897,120	1,897,233	1
N_Bc_pop55	BC1G_01555	Microsatellite	5	65,880	1,066,113	6
N_Bc_pop24	BC1G_05702	Microsatellite	6	1,203,520	1,203,684	3
N_Bc_pop31	BC1G_05733	Microsatellite	6	1,132,149	1,132,260	1
Bc9		Microsatellite	7,2	651,438	27,728,787	4
N_Bc_pop42	BC1G_02801	Microsatellite	7	828,575	828,792	3
HSP5	HSP60	Species Id	7	1,919,752	1,919,977	4
Bc8		Microsatellite	8	1,446,040	1,446,166	2
N_Bc_pop20	BC1G_07837	Microsatellite	8	767,428	767,625	1
N_Bc_pop50	BC1G_14573	Microsatellite	8	78,528	78,698	1
N_Bc_pop52	BC1G_07540	Microsatellite	8	1,501,117	1,501,358	4
N_Bc_pop14	BC1G_12133	Microsatellite	9	1,570,300	1,570,551	1
N_Bc_pop19	BC1G_12993	Microsatellite	9	1,095,632	1,097,796	5
N_Bc_pop71	BC1G_07681	Microsatellite	9	23,238,313	2,328,489	5
N_Bc_pop59	BC1G_01708	Microsatellite	5	2,263,846	2,263,972	7
N_Bc_pop82	BC1G_12477	Microsatellite	5	635,736	635,929	9
mrr3	mrr1	Group S	5	681,986	682,243	37
Cytochrome b	Cytochrome b	Fun resistance	3,8,9 (mitochondrial)	-	-	-
Erg27	Erg27	Fun resistance^y^	3	1,724,223	1,724,411	2
sdhB	sdhB	Fun resistance	1	1,792,746	1,793,044	1
tubA	tubA	Fun resistance	1	2,812,981	2,813,092	1

x Number of individual positional SNPs found within the primer boundaries.

y Fungicide resistance.

**Figure 2:**
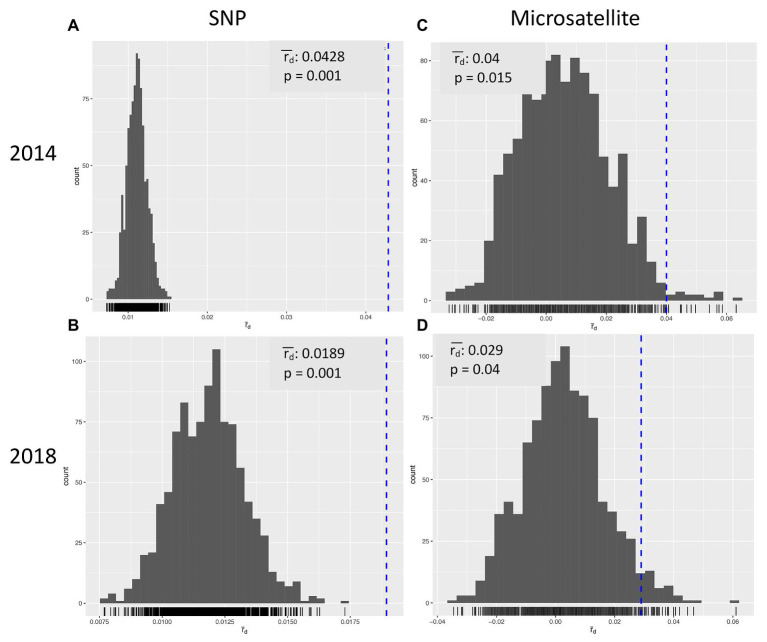
Index of Association for *Botrytis cinerea* isolates collected in Michigan in 2014 **(A,C)** and 2018 **(B,D)** using SNP **(A,B)** or microsatellite **(C,D)** markers.

Multilocus statistics for the population grouped by year showed that the standardized index of association was significant at 0.0428 and 0.0189 for 2014 and 2018, respectively (Table 3; Figures 2A,B). A greater number of unique multilocus genotypes (MLGs) was detected in 2014 than in 2018, but the total number of MLGs between years was similar. A small (1.6%) yet significant (*p* = 0.049) difference was detected between isolates collected in 2014 and those collected in 2018 (Table 4). G’ST was 0.060 and Nei’s unbiased genetic diversity for the two populations was 0.122 (2014) and 0.113 (2018). The greatest SNP contribution was from marker N_Bc_pop90 on chromosome 1 at position 2,341,856 (Supplementary Figure 1).

**Table 3:** Multilocus statistics based on amplicon sequencing for *Botrytis* isolates grouped by year or location of collection.

Pop	N^t^	MLG^u^	eMLG^v^	H^w^	Lambda^x^	Hexp^y^	rbarD^z^
2018	39	39	39	3.66	0.974	0.113	0.0189
2014	42	42	39	3.74	0.976	0.122	0.0428
Total	81	81	39	4.39	0.988	0.118	0.0251
West	20	20	18	3	0.95	0.1125	0.0341
Southwest 1	18	18	18	2.89	0.944	0.0898	0.0735
Southwest 2	25	25	18	3.22	0.96	0.1169	0.0452
Northwest	18	18	18	2.89	0.944	0.0911	0.047
Total	81	81	18	4.39	0.988	0.1181	0.0251

t Number of isolates observed.

u Multilocus genotypes observed.

v Number of expected multilocus genotypes.

w Shannon-Weiner index of MLG diversity (Stoddart and Taylor, 1988).

x Simpson’s index (Simpson, 1949).

y Nei’s unbiased gene diversity (Nei, 1978).

z Standardized index of association.

**Table 4:** AMOVA for *Botrytis* isolates collected from Michigan grouped by year of collection.

	Df^v^	Sum Sq^w^	MSS^x^	% Variability^y^	*p*
Between pop	1	122.897	122.897	1.616264	0.049
Between samples	79	5,640.1228	71.39396	82.84601	0.001
Within samples	81	495.7978	6.12096	15.53773	0.001

v Degrees of freedom.

w Sum of squares.

x Mean sum of squares.

y Percentage of the variability explained by the grouping.

**Figure 3:**
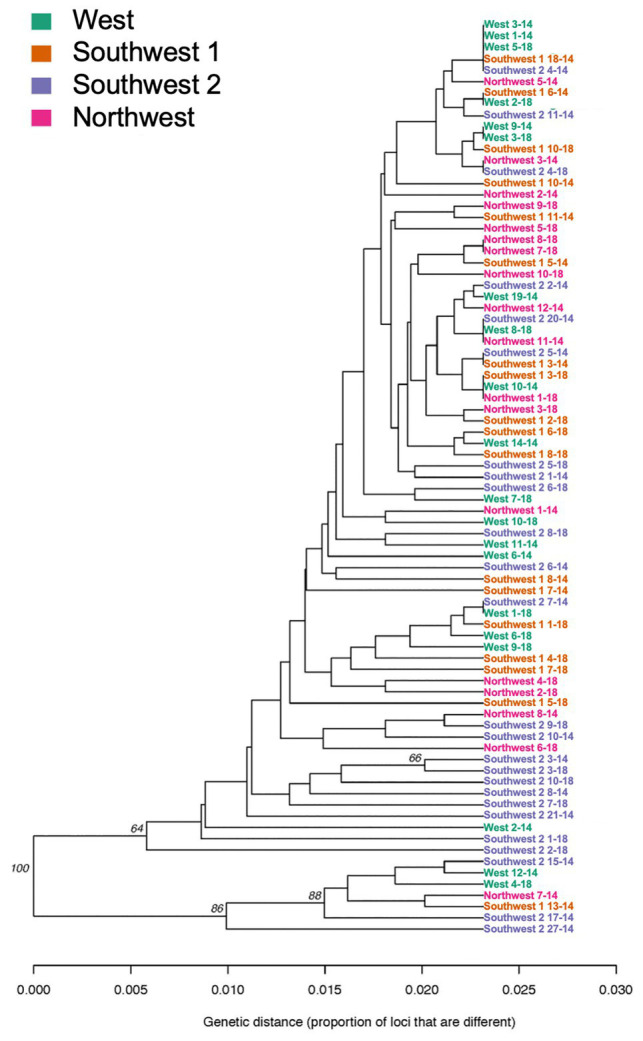
Genetic relatedness of *Botrytis* isolates collected from four locations (West, Southwest 1, Southwest 2, and Northwest) in Michigan based on UPGMA using 496 SNPs.

When grouped by location (West, Southwest 2, Northwest, and Southwest 1), overall G’ST was 0.154. Both the genetic distance tree and AMOVA showed no significant differences based on location among isolates (*p* > 0.05; Figure 3). The standardized index of association for each location was significant, 0.025–0.074. The highest rbarD was detected at Southwest 1, and Southwest 2 had the highest number of MLGs (Table 3). The greatest SNP contribution was from position 1,783,010 located on chromosome 1, which was not located within the aligned primer boundaries for any of the primer sets evaluated (Table 2; Supplementary Figure 2). When grouped by MFR, significant variation (*p* = 0.004, 7.76%) was explained by the populations (Table 5). When grouped by individual fungicide resistances, fluopyram, cyprodinil, and fenhexamid had no significant variation between resistant and sensitive populations. Thiabendazole, iprodione, and boscalid all had moderate variability (7.7–10%) explained between the resistant and sensitive populations (Table 5).

**Table 5:** AMOVA for *Botrytis* isolates collected from Michigan grouped by fungicide resistance using amplicon sequencing.

	Df^v^	Sum Sq^w^	MSS^x^	% Variability^y^	*p*
**Multiple fungicide resistance**					
Between pop	7	846.1156	120.87366	7.76	0.004
Between samples	73	4,916.9042	67.35485	76.88	0.001
Within samples	81	495.7978	6.12096	15.37	0.001
**Thiabendazole**					
Between pop	1	341.9035	341.90351	9.073096	0.001
Between samples	79	5,421.1163	68.62173	76.034211	0.001
Within samples	81	495.7978	6.12096	14.892693	0.001
**Iprodione**					
Between pop	1	226.9774	226.97737	7.769274	0.002
Between samples	79	5,536.0424	70.07649	77.412891	0.001
Within samples	81	495.7978	6.12096	14.817835	0.001
**Boscalid**					
Between pop	1	181.3381	181.33812	10.13	0.013
Between samples	79	5,581.6817	70.6542	75.54	0.001
Within samples	81	495.7978	6.12096	14.33	0.001

v Degrees of freedom.

w Sum of squares.

x Mean sum of squares.

y Percentage of the variability explained by the grouping.

### Microsatellite-Based Diversity

Alleles identified in the population ranged from four to seven, with an average of 5.89 alleles per locus and a total of 53 alleles across all nine markers (Table 6). After clone correction, 56 original multilocus genotypes were identified when grouped by year. Both years had a similar number of MLG identified (30 vs. 27), and the standardized index of association (rbarD) for the populations ranged from 0.040 to 0.029 in 2014 and 2018, respectively (Figures 2C,D). Hedrick’s GST across all markers was 0.044 (Hedrick, 2005; Meirmans and Hedrick, 2011). AMOVA revealed no significant variation explained by grouping the population by year. When grouped by location, genetic diversity was similar across populations (H_exp_ = 0.61–0.64), but rbarD varied widely with slightly negative values for Southwest 2 and West (−0.003 and −0.002, respectively) and a large positive value (0.15) for Southwest 1 (Table 7). However, AMOVA revealed no significant variation explained when grouped by location (*p* = 0.069).

**Table 6:** Marker statistics for *Botrytis* isolates collected in Michigan using microsatellites.

Locus	Allele No	1-D	Hexp	Evenness	GST_Hedrick
BC4	7	0.81	0.82	0.86	0.012158
BC5	5	0.28	0.29	0.45	0.022234
BC8	4	0.49	0.5	0.64	0.074574
BC26	7	0.76	0.78	0.81	−0.042828
BC30	6	0.75	0.76	0.84	0.213684
BC32	5	0.63	0.65	0.79	0.096077
BC37	6	0.68	0.7	0.71	−0.00051
BC54	6	0.62	0.63	0.7	0.019629
BC74	7	0.77	0.78	0.8	0.009837
Mean	5.89	0.64	0.66	0.73	0.04449

**Table 7:** Multilocus statistics based on microsatellites for *Botrytis* isolates grouped by location of collection.

Pop	N^t^	MLG^u^	eMLG^v^	H^w^	lambda^x^	Hexp^y^	rbarD^z^
West	13	13	12	2.56	0.923	0.616	−0.00242
Southwest 1	15	15	12	2.71	0.933	0.644	0.15107
Southwest 2	21	21	12	3.04	0.952	0.637	−0.00376
Northwest	12	12	12	2.48	0.917	0.614	0.07412
Total	61	56	11.7	3.97	0.979	0.646	0.04254

t Number of isolates observed.

u Multilocus genotypes observed.

v Number of expected multilocus genotypes.

w Shannon-Weiner index of MLG diversity (Stoddart and Taylor, 1988).

x Simpson’s index (Simpson, 1949).

y Nei’s unbiased gene diversity (Nei, 1978).

z Standardized index of association.

When grouped by fungicide resistance (individual or MFR), no significant variability was detected between resistant and sensitive populations for cyprodinil, iprodione, or fenhexamid. MFR was grouped into categories designated 3 (0–3), 4, and 5 (5–7) because insufficient numbers of isolates had resistance to 0, 1, 2, 6, or 7 fungicides. AMOVA results indicated significant variability in the population was explained when grouped by MFR (0–7) or MFR categories (3, 4, 5; 3.7% at *p* = 0.037 and 4.4% at *p* = 0.012, respectively; Table 8). When grouped into resistant or sensitive categories for thiabendazole or boscalid, 14% of the population variability (*p* = 0.001) was explained (Table 8). When grouped by fluopyram resistance or sensitive groupings, 4.4% of the variability was explained (*p* = 0.026).

**Table 8:** AMOVA for *Botrytis* isolates collected from Michigan grouped by fungicide resistance using microsatellites.

	Df^v^	Sum Sq^w^	MSS^x^	% Variability^y^	*p*
**Multiple fungicide resistance**					
Between samples	2	16.30676	8.15338	4.398508	0.012
Within samples	55	244.02321	4.436786	95.601492	
**Thiabendazole**					
Between samples	1	19.17995	19.179954	14.04678	0.001
Within samples	54	233.69633	4.32771	85.95322	
**Boscalid**					
Between samples	1	18.14639	18.146391	14.17922	0.001
Within samples	58	345.84913	5.962916	85.82078	
**Fluopyram**					
Between samples	1	8.399909	8.399909	4.327576	0.026
Within samples	55	248.778799	4.523251	95.672424	

v Degrees of freedom.

w Sum of squares.

x Mean sum of squares.

y Percentage of the variability explained by the grouping.

### Comparison of Single-Nucleotide Polymorphisms and Microsatellite Markers

Both SNP and microsatellite markers were able to identify significant population differentiation and genetic diversity in *Botrytis* isolates from Michigan. A greater number of MLG groups was identified across the total population and within each location with SNP markers compared with microsatellites (Tables 3 and 7). Both marker systems demonstrated significant linkage among markers and genotypes across both years for rbarD suggesting that populations persist across years (Figure 2). For instance, MFR groupings were significant using both microsatellites and SNPs; however, SNP markers explained a greater proportion of the variation by this grouping than microsatellites (7.76% compared with 3.68%, respectively.) Thiabendazole and boscalid resistance groupings were consistently associated with significant population structure. Population structure associated with fluopyram resistance, a FRAC 7 similar boscalid, was only significant using the microsatellite markers (4.3% at *p* = 0.026).

## Discussion

In this study, we evaluated SNP and microsatellite markers for their ability to describe genetic diversity and population structure in *B. cinerea* using isolates collected from Michigan vineyards. *B. cinerea* is a globally distributed pathogen with a broad host range and widespread fungicide resistance. Thousands of studies across the globe have used morphological, genic, microsatellite, and other PCR-based markers to characterize genetic diversity and population structure of *Botrytis* spp. The pathogen is genetically diverse with closely related morphologically similar species that can be found on the same host or tissue complicate diversity studies (Walker et al., 2011; Li et al., 2012; Saito et al., 2016; Garfinkel et al., 2017; Rupp et al., 2017b; Hu et al., 2018; Harper et al., 2019). While it is widely accepted that population structure exists within *Botrytis*, the degree and factors by which populations can be differentiated has not been consistent. Depending on the populations and marker systems tested, population structures associated with continent, host, year, and fungicide resistance have all been reported at varying levels of differentiation and significance (Fournier and Giraud, 2008; Muñoz and Moret, 2010; Walker et al., 2015; Delong et al., 2020; Rupp et al., 2017a,b). This variability could be, in part, caused by regional differences in *Botrytis* population composition. Fungicide spray programs and the resulting resistance have repeatedly been shown to play a large role in *Botrytis* field population composition, but smaller differences caused by host, season, or marker resolution may also play a role (Wessels et al., 2016; Kozhar et al., 2020; Testempasis et al., 2020).

Microsatellites have historically been used to assess genetic diversity and population structure because of their widespread accessibility, requiring no prior known sequence information, highly specialized equipment, or software to analyze. As sequencing technologies have become more affordable, SNP-based assessment of genetic diversity and population structure has become more prevalent (Loera-Sanchez et al., 2019; Sato et al., 2019; Li et al., 2020; Weldon et al., 2020). Using sequencing data has multiple advantages to microsatellites, primarily that no prior sequence information is required, marker transferability is not a concern, and sequence information can provide higher resolution for individuals (Helyar et al., 2011; Du et al., 2019). However, studies have shown that for organisms with larger genomes, well-designed microsatellites are often more effective than small (<400) numbers of SNPs at characterizing differentiation (Vali et al., 2008; Müller et al., 2015; Lemopoulos et al., 2018). However, *Botrytis*, like many fungal organisms, has a small genome (<45 Mb) and may not require large (>400) numbers of SNPs for accurately differentiating populations (Abbott et al., 2010; Tsykun et al., 2017; Van Kan et al., 2017). This could be achieved either through whole genome sequencing or reduced representation sequencing. However, whole genome sequencing may be cost prohibitive for large numbers of isolates. Amplicon sequencing, compared with GBS, has the added advantage of targeting known regions allowing for comparison of genes of interest for isolates across different studies. As more *Botrytis* genomes are sequenced, better target sequences (semi-conserved with high diversity across isolates, fungicide resistance genes, etc.) can be selected to improve detection.

In this study, we identified 496 SNPs from 52 amplicons and nine microsatellites to characterize 82 and 74 isolates, respectively. Both types of markers were able to identify genetic diversity and population structure across the shared isolates. High levels of clonality were observed between years, suggesting that these populations can be persistent over multiple years in perennial crops. In our study, *Botrytis* isolates were collected in 2014 and 2018. When comparing genetic diversity between the two marker systems, the 496 SNPs were only able to distinguish an additional two to three MLGs for the locations with the highest number (Southwest 1 and Southwest 2), but with the two lower MLG locations (West and Northwest), more than six additional MLGs were detected. While both types of markers were able to differentiate population structure at the multiple fungicide resistance level, thiabendazole and boscalid resistance, neither marker system was able to detect significant differentiation based on location similar to other *Botrytis* studies (Hu et al., 2018; Delong et al., 2020; Testempasis et al., 2020). Minor differences associated with region were detected with microsatellite markers, but were not significant at the *p* = 0.05 level. Significant differentiation based on single fungicide resistances and year of collection were also detected, but differed, between the two marker systems. This could be, in part, due to specific known fungicide resistance-associated loci being included with the SNP data set and not necessarily in the microsatellites. Yet microsatellite markers were able to differentiate population structure associated with a similar number of fungicides as the SNP markers. Resistance to multiple fungicides and iprodione each explained approximately 7% of the variability observed, but boscalid resistance explained the majority of the differentiation (10% with SNPs and 14% with microsatellites). This is similar to other studies showing that fungicide resistance may be a driving factor in *Botrytis* population structure in agricultural systems (Kozhar et al., 2020). In summary, microsatellites and SNP markers were both effective at identifying population structure associated with major factors (e.g., fungicide resistance) in *Botrytis*. However, as populations with greater numbers of individuals are evaluated, SNP markers will likely be more cost effective and useful for identifying closely related species and minor factors associated with population structure.

## Data Availability Statement

The datasets generated for this study can be found in the NCBI SRA repository (https://www.ncbi.nlm.nih.gov/biosample/18170356).

## Author Contributions

RN conceived of experiment, analyzed the data, and contributed to writing. JD collected and analyzed the data and contributed to writing. NA and SS collected the data and contributed to the experimental design and writing. TM and SA contributed to the experiment design and writing. All authors contributed to the article and approved the submitted version.

### Conflict of Interest

The authors declare that the research was conducted in the absence of any commercial or financial relationships that could be construed as a potential conflict of interest.

## Footnotes

^1^https://usegalaxy.org/

## Supplementary Material

Supplementary Figure 1 SNP loading plot showing the contribution of different SNPs when grouped by year of collection (2014 vs. 2018) for isolates of *B. cinerea* from Michigan vineyards.

Supplementary Figure 2 SNP loading plot showing the contribution of different SNPs when grouped by location of collection (West, Southwest1, Southwest2, and Northwest) in Michigan.
