# Supplementary figures and images for: Population Genetic Analyses of Botrytis cinerea Isolates From Michigan Vineyards Using a High-Throughput Marker System Approach
Source: Front Microbiol. 2021 Apr 20;12:660874. doi: 10.3389/fmicb.2021.660874 (PMC8093758; doi:10.3389/fmicb.2021.660874)

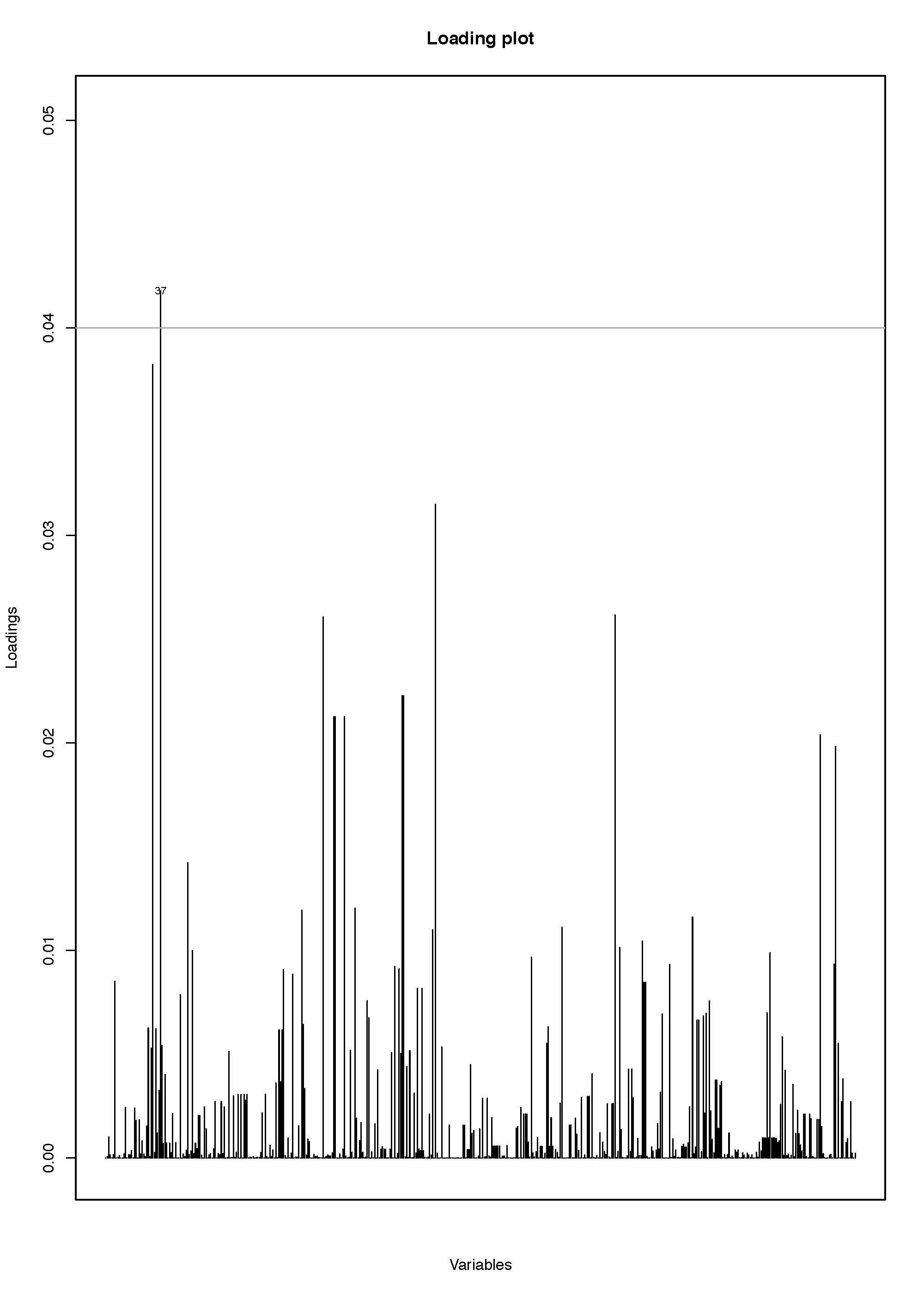

Supplement: Supplementary Figure 1 — SNP loading plot showing the contribution of different SNPs when grouped by year of collection (2014 vs. 2018) for isolates of B. cinerea from Michigan vineyards. [file Image_1.JPEG]

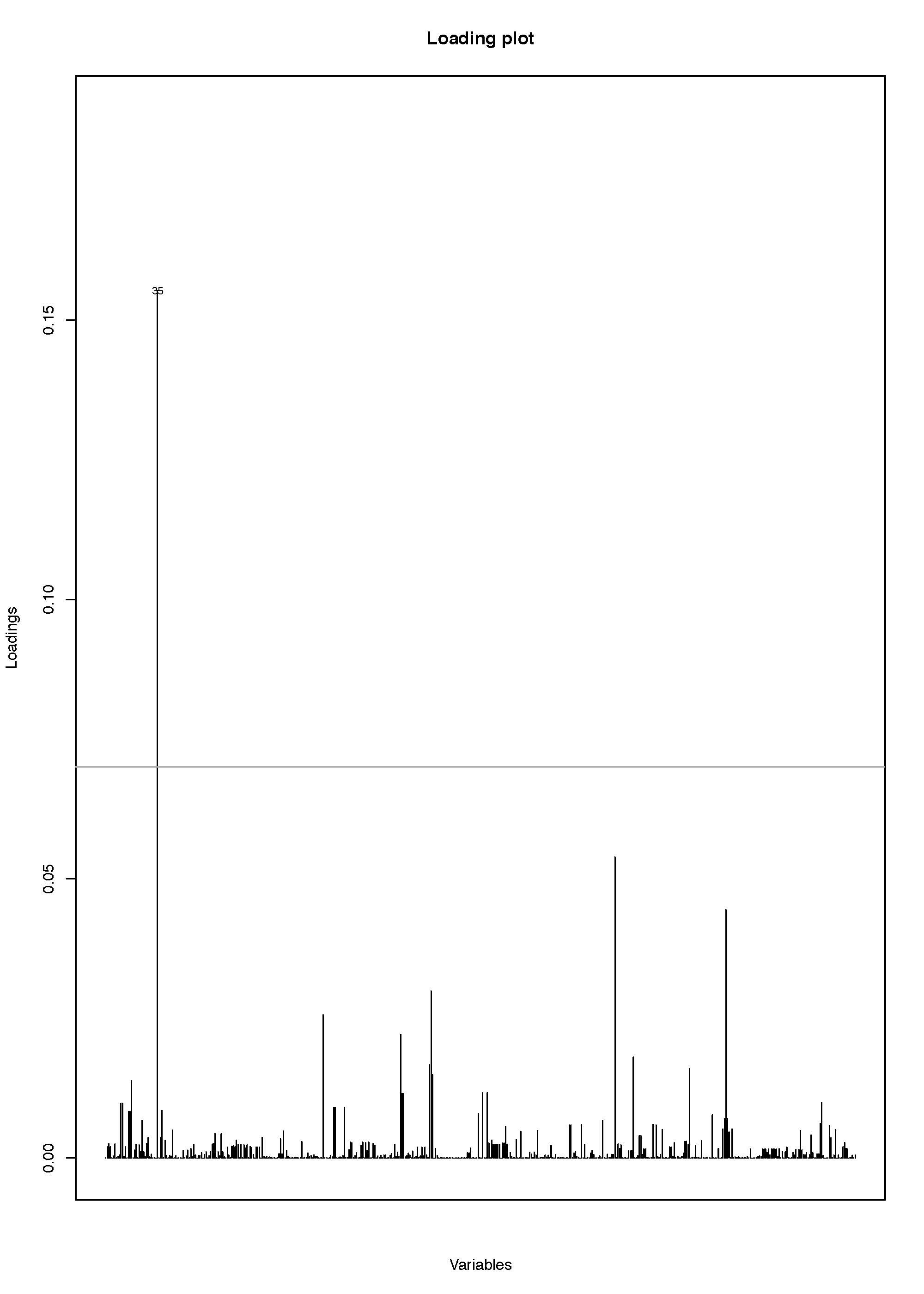

Supplement: Supplementary Figure 2 — SNP loading plot showing the contribution of different SNPs when grouped by location of collection (West, Southwest1, Southwest2, and Northwest) in Michigan. [file Image_2.JPEG]
